# Development and Validation of a Predictive Model to Evaluate the Risk of Bone Metastasis in Kidney Cancer

**DOI:** 10.3389/fonc.2021.731905

**Published:** 2021-11-25

**Authors:** Shengtao Dong, Hua Yang, Zhi-Ri Tang, Yuqi Ke, Haosheng Wang, Wenle Li, Kang Tian

**Affiliations:** ^1^ Department of Bone and Joint, First Affiliated Hospital, Dalian Medical University, Dalian, China; ^2^ Department of Spine Surgery, Second Affiliated Hospital of Dalian Medical University, Dalian, China; ^3^ Department of Otolaryngology, Head and Neck Surgery, Second Affiliated Hospital of Dalian Medical University, Dalian, China; ^4^ School of Physics and Technology, Wuhan University, Wuhan, China; ^5^ Department of Orthopaedics Surgery, Second Affiliated Hospital of Dalian Medical University, Dalian, China; ^6^ Orthopaedic Medical Center, The Second Hospital of Jilin University, Changchun, China; ^7^ Department of Orthopedics, Xianyang Central Hospital, Xianyang, China; ^8^ Clinical Medical Research Center, Xianyang Center Hospital, Xianyang, China

**Keywords:** renal cell carcinoma, bone metastasis, nomogram, web calculator, predictive model

## Abstract

**Background:**

Bone is a common target of metastasis in kidney cancer, and accurately predicting the risk of bone metastases (BMs) facilitates risk stratification and precision medicine in kidney cancer.

**Methods:**

Patients diagnosed with kidney cancer were extracted from the Surveillance, Epidemiology, and End Results (SEER) database to comprise the training group from 2010 to 2017, and the validation group was drawn from our academic medical center. Univariate and multivariate logistic regression analyses explored the statistical relationships between the included variables and BM. Statistically significant risk factors were applied to develop a nomogram. Calibration plots, receiver operating characteristic (ROC) curves, probability density functions (PDF), and clinical utility curves (CUC) were used to verify the predictive performance. Kaplan-Meier (KM) curves demonstrated survival differences between two subgroups of kidney cancer with and without BMs. A convenient web calculator was provided for users *via* “shiny” package.

**Results:**

A total of 43,503 patients were recruited in this study, of which 42,650 were training group cases and 853 validation group cases. The variables included in the nomogram were sex, pathological grade, T-stage, N-stage, sequence number, brain metastases, liver metastasis, pulmonary metastasis, histological type, primary site, and laterality. The calibration plots confirmed good agreement between the prediction model and the actual results. The area under the curve (AUC) values in the training and validation groups were 0.952 (95% CI, 0.950–0.954) and 0.836 (95% CI, 0.809–0.860), respectively. Based on CUC, we recommend a threshold probability of 5% to guide the diagnosis of BMs.

**Conclusions:**

The comprehensive predictive tool consisting of nomogram and web calculator contributes to risk stratification which helped clinicians identify high-risk cases and provide personalized treatment options.

## Introduction

Kidney cancer is one of the 10 most oncologic diseases to plague the USA (1, 2). Kidney cancer has historically been considered in general terms as a single disease. As continued exploration at the genetic level has shown that it is composed of several different types of cancer, characterized by different mutated genes corresponding to different histologies, clinical processes, and responses to treatment (3), renal cell carcinoma (RCC) accounts for 90% of kidney cancer with a global annual incidence and mortality rate of approximately 400,000 and 175,000, respectively (4, 5). RCC is a highly heterogeneous tumor and has an obvious tendency to metastasize distantly. Given this feature, 30% of patients will be diagnosed with metastasis even after aggressive treatment of the primary tumor (6). Bone metastases (BMs), with a 40% incidence rate, are a common mechanism of metastasis and have been identified as a major prognostic risk factor associated with poor survival in patients with metastasic RCC (mRCC) (6–8). The frequent sites of colonization by mRCC cells are the proximal extremities and pelvis, manifesting as bone pain, pathological fractures, and hypercalcemia. When spine is involved, devastating paraplegia can occur due to the spinal cord compression. It has been shown previously that metastatic tumors affect bone turnover differently, and radiographic images of bone metastatic tumors from mRCC are osteolytic or osteoclastic due to an imbalance between osteoclasts and osteoblasts mediated by tumor cells. Osteoclasts often increase their activity because of the upregulation of kappa-B ligand (RANKL) induced by mRCC (7). As a result, the osteoclast inhibitor bisphosphonates and the RANKL blocker denosumab are widely used to treat BMs from mRCC.

However, some of the treatment outcomes are still unsatisfactory. There is still a lack of standard treatment protocols or guidelines, most treatments focus solely on improving skeletal adverse events in mRCC with BMs (9). Only a few retrospective studies and case reports have confirmed that early diagnosis and timely wide resections are critical in clinical management for patients with mRCC (6, 10–13). Therefore, the development of a predictive model to assess the risk of BMs in mRCC is an important part of achieving precision medicine, which includes more aggressive selection of surgical periods, enhanced surveillance, and regular bone scans.

In contrast to the high threshold of background knowledge required that clinicians need to obtain in order to use artificial intelligence, a simplicity and intuitiveness of nomogram can provide the same insightful analysis to help clinicians decide the clinical treatment. Several literatures have developed nomograms to predict prognosis targeting BMs from mRCC (14, 15). However, the current study constructs the first predictive model to predict risk factors for BMs, facilitating clinicians in making individualized clinical decisions and assessing patients’ long-term prognosis. We extracted kidney cancer patient data from the Surveillance, Epidemiology, and End Results (SEER) database and verified it with an independent validation dataset to mitigate the regional limitations of this study to the extent possible.

## Methods

### Study Design and Participants

Based on the SEER database, we extracted patients diagnosed with kidney cancer from 2010 to 2017 as the training group through SEER*STAT software (version 8.3.5). Validation group of patients from a large academic medical center and the time span matched to the SEER database.

The following inclusion criteria were practiced in the training group: (1) patients with primary kidney cancer (International Classification of Diseases for Oncology ICD-O. 8120/3, 8130/3, 8260/3, 8310/3, 8312/3, 8317/3) diagnosed between January 1, 2010 and December 31, 2017 and (2) diagnosis was based on surviving patients. Included histological subtypes were clear-cell RCC, papillary, chromophobe, and any kidney cancer. Cancer-specific survival (CSS) was defined based on the SEER mortality codes. Further inclusion criteria practiced in validation groups include the following: (1) the patient was older than 18 years, (2) sufficient radiological outcomes and pathological biopsy results during follow-up to determine if metastases are developing, and (3) the follow-up data were obtained until December 31, 2020.

Cases meeting the following criteria were excluded, as follows: (1) patients younger than 18, (2) multiple primary tumors, (3) unavailable demographic characteristics (age and sex), (4) unavailable tumor information (histological type, pathological grade, laterality, TNM stage, and sequence number), (5) diagnosis was from cadavers, (6) without or with unknown BMs and survival time, and (7) cause of death unrelated to kidney cancer or unknown.

Histological subtype was determined according to the International Classification of Diseases for Oncology code. Oncology staging was determined according to the 7th TNM classification of the American Joint Committee on Cancer.

According to the standard NAACCR terms, patients were assigned to two groups: one group were diagnosed with only one primary tumor and the other group were diagnosed with more than one tumor (16).

This study was approved by the institutional ethics committee.

### Data Collection

All data for the training group were obtained from the SEER database, including the year of diagnosis, age at diagnosis, sex, pathological grade, TNM stage, histological type, primary site, laterality, and metastasis. Diagnosis in the independent validation group was completed separately by two pathologists in a blinded manner, and a senior pathologist performed review and final diagnosis of controversial patients. If any abnormalities are found, we recommend patients undergo a whole-body CT scan to help identify metastatic lesions. A radionuclide bone scan is used to evaluate the presence of bone metastasis, and PET-CT is also used to exclude insidious tumor metastasis. Diagnosis of suspected metastasis relied on pathological biopsy of the metastatic site. Follow-up documentation consisted mainly of remote follow-up and outpatient review. The primary endpoint event was the presence of BMs, and sub-endpoint event was survival time (as of death due to kidney or last follow-up). All validation data were obtained from our medical electronic records.

### Statistical Analysis

Independent Samples *t*-tests and ordinary Chi-square tests were performed to analyze the characteristics of all included patients. In the training group, we screened the results of statistically different univariate logistic regression analyses for multivariate analysis. Furthermore, validated independent risk factors were used to construct a nomogram to assess the odds of BMs in patients with RCC. The predictive performance of this nomogram was explored by receiver operating characteristic (ROC) curves, calibration plots, probability density functions (PDF), and clinical utility curve (CUC) (17). The OS of mRCC patients with BMs was demonstrated by Kaplan-Meier curves. Statistical analysis was performed using SPSS (version 20.0, Chicago, IL, USA). *p*-values <0.05 were considered statistically significant. R software (version 4.0.5, https://www.r-project.org/) was applied for developing predictive model using “rms” package and the “shiny” package to establish a nomogram and web calculator, respectively.

## Results

### Included Patients

A total of 43,503 patients with kidney cancer were included in the present study. The SEER database provided 42,650 available patients for the training group. After screening out 279 patients (86 with multiple tumors, 75 lacking survival time due to loss to follow-up, 65 dying from nontumor factors, 33 lacking records of metastasis, and 20 with unavailable pathological diagnoses), 853 patients from the Second Affiliated Hospital of Dalian Medical University were grouped as an independent validation group. Older males were the predominant patient population. There were statistical differences in race (*p* < 0.001), marital status (*p* < 0.001), primary site (*p* < 0.001), laterality (*p* = 0.001), grade (*p* < 0.001), histology (*p* = 0.003), T-stage (*p* = 0.015), N-stage (*p* = 0.016), surgery (*p* < 0.001), chemotherapy (*p* = 0.005), bone metastasis (*p* < 0.001), and liver metastasis (*p* < 0.026) of the training and validation groups. These differences may be due to demographic differences and healthcare disparities between the USA and China. The detailed clinical characteristics of the patients are demonstrated in **Table 1**. **Table 2** compares the cohort differences between the BM and non-BM groups.

**Table 1:** Comparison of patients in the training group and validation group.

Characteristics	Level	Training group (*N* = 42,650)	Validation group (*N* = 853)	*p*-value
Age [mean (SD)]		63.50 (13.07)	63.90 (13.10)	0.38
Sex (%)	Female	15,079 (35.4)	309 (36.2)	0.624
	Male	27,571 (64.6)	544 (63.8)	
Race (%)	White	33,344 (78.2)	0 (0.0)	<0.001
	Black	5,389 (12.6)	0 (0.0)	
	Chinese	512 (1.2)	853 (100.0)	
	Other	3,405 (8.0)	0 (0.0)	
Marital (%)	Married	25,058 (58.7)	561 (65.8)	<0.001
	Unmarried	15,469 (36.3)	292 (34.2)	
	Unknown	2,123 (5.0)	0 (0.0)	
Sequence number (%)	More than 1	14,030 (32.9)	259 (30.4)	0.128
	1 primary only	28,620 (67.1)	594 (69.6)	
Primary Site (%)	Kidney	40,566 (95.1)	763 (89.4)	<0.001
	Renal pelvis	2,084 (4.9)	90 (10.6)	
Laterality (%)	Right	21,495 (50.4)	424 (49.7)	0.001
	Left	21,068 (49.4)	422 (49.5)	
	Other	87 (0.2)	7 (0.8)	
Grade (%)	Well	3,387 (7.9)	79 (9.2)	<0.001
	Moderately	14,651 (34.4)	313 (36.7)	
	Poorly	8,915 (20.9)	254 (29.8)	
	Undifferentiated	3,337 (7.8)	69 (8.1)	
	Unknown	12,360 (29.0)	138 (16.2)	
Histology (%)	Clear cell adenocarcinoma	22,616 (53.0)	470 (55.1)	0.003
	Renal cell carcinoma	7,823 (18.4)	149 (17.5)	
	Papillary adenocarcinoma	5,278 (12.4)	78 (9.1)	
	Renal cell carcinoma, chromophobe type	2,231 (5.2)	50 (5.9)	
	Transitional cell carcinoma, NOS	1,142 (2.7)	34 (4.0)	
	Papillary transitional cell carcinoma	1,033 (2.4)	30 (3.5)	
	Other	2,527 (5.9)	42 (4.9)	
T (tumor invasion, %)	T1	27,898 (65.4)	513 (60.1)	0.015
	T2	4,247 (10.0)	104 (12.2)	
	T3	8,428 (19.7)	186 (21.8)	
	T4	1,143 (2.7)	24 (2.8)	
	TX	934 (2.2)	26 (3.1)	
N (regional lymph node, %)	N0	38,388 (90.0)	753 (88.3)	0.016
	N1	2,431 (5.7)	66 (7.7)	
	N2	199 (0.5)	0 (0.0)	
	NX	1,632 (3.8)	34 (4.0)	
Tumor Size [mean (SD)]		51.65 (41.15)	52.01 (37.19)	0.797
Surgery (%)	Radical nephrectomy	15,717 (36.9)	293 (34.3)	<0.001
	Complete/total/simple nephrectomy	3,814 (9.0)	91 (10.7)	
	Partial/subtotal nephrectomy/partial ureterectomy	12,040 (28.2)	206 (24.2)	
	Local tumor destruction	2,102 (4.9)	55 (6.4)	
	Local tumor excision	894 (2.1)	33 (3.9)	
	Any nephrectomy	317 (0.7)	7 (0.8)	
	No surgery	7,766 (18.2)	168 (19.7)	
Radiation (%)	Yes	1,642 (3.8)	33 (3.9)	1
	None/Unknown	41,008 (96.2)	820 (96.1)	
Chemotherapy (%)	Yes	3,536 (8.3)	94 (11.0)	0.005
	None/Unknown	39,114 (91.7)	759 (89.0)	
	Yes	2,412 (5.7)	61 (7.2)	
System treatment (%)	None/Unknown	40,238 (94.3)	792 (92.8)	0.073
Bone metastasis (%)	Yes	1,951 (4.6)	67 (7.9)	<0.001
	No	40,699 (95.4)	786 (92.1)	
Brain metastasis (%)	Yes	540 (1.3)	18 (2.1)	0.057
	No	42,058 (98.6)	835 (97.9)	
	Unknown	52 (0.1)	0 (0.0)	
	Yes	1,045 (2.5)	32 (3.8)	
Liver metastasis (%)	No	41,533 (97.4)	821 (96.2)	0.026
	Unknown	72 (0.2)	0 (0.0)	
Pulmonary metastasis (%)	Yes	3,171 (7.4)	69 (8.1)	0.513
	No	39,479 (92.6)	784 (91.9)	
Survival time [mean (SD)]		39.06 (30.69)	37.13 (30.82)	0.068

Other, less than 1,000 cases.

**Table 2:** Comparison of patients with or without BMs.

Characteristics	Level	NBMs (*N* = 41,485)	BMs (*N* = 2,018)	*p*-value
Category (%)	Training group	40,699 (98.1)	1,951 (96.7)	<0.001
	Validation group	786 (1.9)	67 (3.3)	
Age [mean (SD)]		63.39 (13.08)	65.90 (12.52)	<0.001
Sex (%)	Female	14,792 (35.7)	596 (29.5)	<0.001
	Male	26,693 (64.3)	1,422 (70.5)	
Race (%)	White	31,761 (76.6)	1,583 (78.4)	<0.001
	Black	5,193 (12.5)	196 (9.7)	
	Chinese	1,270 (3.1)	95 (4.7)	
	Other	3,261 (7.9)	144 (7.1)	
Marital (%)	Married	24,484 (59.0)	1,135 (56.2)	<0.001
	Unmarried	14,947 (36.0)	814 (40.3)	
	Unknown	2,054 (5.0)	69 (3.4)	
Sequence number (%)	More than 1	13,874 (33.4)	415 (20.6)	<0.001
	1 primary only	27,611 (66.6)	1,603 (79.4)	
Primary site (%)	Kidney	39,392 (95.0)	1,937 (96.0)	0.043
	Renal pelvis	2,093 (5.0)	81 (4.0)	
Laterality (%)	Right	20,945 (50.5)	974 (48.3)	<0.001
	Left	20,473 (49.4)	1,017 (50.4)	
	Other	67 (0.2)	27 (1.3)	
Grade (%)	Well	3,447 (8.3)	19 (0.9)	<0.001
	Moderately	14,812 (35.7)	152 (7.5)	
	Poorly	8,873 (21.4)	296 (14.7)	
	Undifferentiated	3,165 (7.6)	241 (11.9)	
	Unknown	11,188 (27.0)	1,310 (64.9)	
Histology (%)	Clear cell adenocarcinoma	22,226 (53.6)	860 (42.6)	<0.001
	Renal cell carcinoma	7,274 (17.5)	698 (34.6)	
	Papillary adenocarcinoma	5,290 (12.8)	66 (3.3)	
	Renal cell carcinoma, chromophobe type	2,256 (5.4)	25 (1.2)	
	Transitional cell carcinoma, NOS	1,087 (2.6)	89 (4.4)	
	Papillary transitional cell carcinoma	1,052 (2.5)	11 (0.5)	
	Other	2,300 (5.5)	269 (13.3)	
T (tumor invasion, %)	T1	27,835 (67.1)	576 (28.5)	<0.001
	T2	3,993 (9.6)	358 (17.7)	
	T3	8,004 (19.3)	610 (30.2)	
	T4	950 (2.3)	217 (10.8)	
	TX	703 (1.7)	257 (12.7)	
N (regional lymph node, %)	N0	37,963 (91.5)	1,178 (58.4)	<0.001
	N1	1,911 (4.6)	586 (29.0)	
	N2	174 (0.4)	25 (1.2)	
	NX	1,437 (3.5)	229 (11.3)	
Tumor size [mean (SD)]		50.37 (40.29)	78.07 (47.54)	<0.001
Surgery (%)	Radical nephrectomy	15,532 (37.4)	478 (23.7)	<0.001
	Complete/total/simple nephrectomy	3,838 (9.3)	67 (3.3)	
	Partial/subtotal nephrectomy/partial ureterectomy	12,211 (29.4)	35 (1.7)	
	Local tumor destruction	2,148 (5.2)	9 (0.4)	
	Local tumor excision	919 (2.2)	8 (0.4)	
	Any nephrectomy	308 (0.7)	16 (0.8)	
	No surgery	6,529 (15.7)	1,405 (69.6)	
Radiation (%)	Yes	594 (1.4)	1,081 (53.6)	<0.001
	None/Unknown	40,891 (98.6)	937 (46.4)	
Chemotherapy (%)	Yes	2,614 (6.3)	1,016 (50.3)	<0.001
	None/Unknown	38,871 (93.7)	1,002 (49.7)	
System treatment (%)	Yes	1,961 (4.7)	512 (25.4)	<0.001
	None/Unknown	39,524 (95.3)	1,506 (74.6)	
Brain metastasis (%)	Yes	339 (0.8)	219 (10.9)	<0.001
	No	41,129 (99.1)	1,764 (87.4)	
	Unknown	17 (0.0)	35 (1.7)	
Liver metastasis (%)	Yes	668 (1.6)	409 (20.3)	<0.001
	No	40,785 (98.3)	1,569 (77.8)	
	Unknown	32 (0.1)	40 (2.0)	
Pulmonary metastasis (%)	Yes	2,209 (5.3)	1,031 (51.1)	<0.001
	No	39,276 (94.7)	987 (48.9)	
Survival time [mean (SD)]		40.27 (30.64)	13.42 (18.25)	<0.001

NBMs, no bone metastasis; BMs, bone metastasis; Other, less than 1,000 cases.

### Correlation of Variable With BMs

Independent risk factors for BMs were obtained by univariate and multivariate logistic regression analyses. Univariate analysis showed that age, sex, race, marital, sequence number, primary site, laterality, grade, histology, T-stage, N-stage, tumor size, and brain/liver/pulmonary metastasis were associated with BMs. Variables including sex, primary site, laterality, pathological grade, histology, T-stage, N-stage, sequence number, and brain/liver/pulmonary metastasis were indicated by multivariate analysis to influence the endpoint outcome events (**Table 3**).

**Table 3:** Relationship between variables and BMs by univariate and multivariate logistic regression analyses.

Characteristics	Univariate logistics regression			Multivariable logistics regression		
	OR	95% CI	*p*-value	OR	95% CI	*p*-value
Age	1.02	1.01–1.02	<0.001	1	1–1.01	0.052
Sex						
Female	Ref	Ref	Ref	Ref	Ref	Ref
Male	1.31	1.19–1.45	<0.001	1.23	1.1–1.38	<0.001
Race						
Black	Ref	Ref	Ref	Ref	Ref	Ref
White	1.32	1.14–1.54	<0.001	1.11	0.93–1.32	0.242
Chinese	1.53	1.02–2.30	0.04	1.28	0.81–2.01	0.293
Other	1.17	0.94–1.46	0.161	0.91	0.71–1.18	0.485
Marital						
Married	Ref	Ref	Ref	Ref	Ref	Ref
Unmarried	1.19	1.08–1.3	<0.001	1.04	0.93–1.16	0.507
Unknown	0.74	0.58–0.95	0.016	0.77	0.58–1.01	0.063
Sequence number						
More than 1	Ref	Ref	Ref	Ref	Ref	Ref
1 primary only	1.93	1.73–2.16	<0.001	1.49	1.31–1.69	<0.001
Primary site						
Kidney	Ref	Ref	Ref	Ref	Ref	Ref
Renal pelvis	0.77	0.61–0.97	0.029	0.44	0.27–0.73	0.002
Laterality						
Left	Ref	Ref	Ref	Ref	Ref	Ref
Right	0.94	0.85–1.03	0.16	1.03	0.93–1.14	0.611
Other	7.78	4.84–12.5	<0.001	2.36	1.32–4.19	0.004
Grade						
Moderately	Ref	Ref	Ref	Ref	Ref	Ref
Well	0.51	0.31–0.85	0.009	0.58	0.35–0.96	0.036
Poorly	3.17	2.58–3.89	<0.001	1.96	1.59–2.43	<0.001
Undifferentiated	7.62	6.16–9.41	<0.001	3.02	2.39–3.83	<0.001
Unknown	11.8	9.91–14.05	<0.001	5.33	4.39–6.47	<0.001
Histology						
Transitional cell carcinoma	Ref	Ref	Ref	Ref	Ref	Ref
Papillary transitional cell carcinoma	0.12	0.06–0.24	<0.001	0.34	0.17–0.67	0.002
Papillary adenocarcinoma	0.15	0.11–0.21	<0.001	0.31	0.18–0.51	<0.001
Clear-cell adenocarcinoma	0.47	0.38–0.6	<0.001	0.8	0.51–1.25	0.317
Renal cell carcinoma	1.18	0.93–1.49	0.167	0.71	0.45–1.12	0.138
Renal cell carcinoma, chromophobe type	0.14	0.09–0.21	<0.001	0.22	0.12–0.41	<0.001
Other	1.43	1.11–1.85	0.006	0.95	0.61–1.5	0.838
T (tumor invasion)						
T1	Ref	Ref	Ref	Ref	Ref	Ref
T2	4.36	3.8–5.01	<0.001	2.01	1.69–2.4	<0.001
T3	3.76	3.34–4.24	<0.001	1.72	1.47–2	<0.001
T4	11.17	9.41–13.27	<0.001	1.99	1.59–2.5	<0.001
TX	18.44	15.6–21.79	<0.001	2.71	2.19–3.36	<0.001
N (regional lymph node)						
N0	Ref	Ref	Ref	Ref	Ref	Ref
N1	10.14	9.07–11.32	<0.001	2.16	1.88–2.48	<0.001
N2	4.73	3.1–7.23	<0.001	3.11	1.78–5.43	<0.001
NX	5.21	4.47–6.08	<0.001	1.67	1.37–2.02	<0.001
Tumor size	1.01	1.01–1.01	<0.001	1.00	1.00–1.00	0.741
Brain metastasis						
No	Ref	Ref	Ref	Ref	Ref	Ref
Yes	15.05	12.58–18.01	<0.001	2.36	1.91–2.92	<0.001
Unknown	48.7	27.23–87.10	<0.001	3.98	1.95–8.11	<0.001
Liver metastasis						
No	Ref	Ref	Ref	Ref	Ref	Ref
Yes	15.96	13.95–18.28	<0.001	2.34	1.99–2.75	<0.001
Unknown	32.97	20.66–52.63	<0.001	2.91	1.61–5.27	<0.001
Pulmonary metastasis						
No	Ref	Ref	Ref	Ref	Ref	Ref
Yes	18.9	17.12–20.86	<0.001	4.31	3.81–4.88	<0.001

OR, odds ratio; 95% CI, 95% confidence interval.

### Develop and Validate Predictive Models

**Figure 1:**
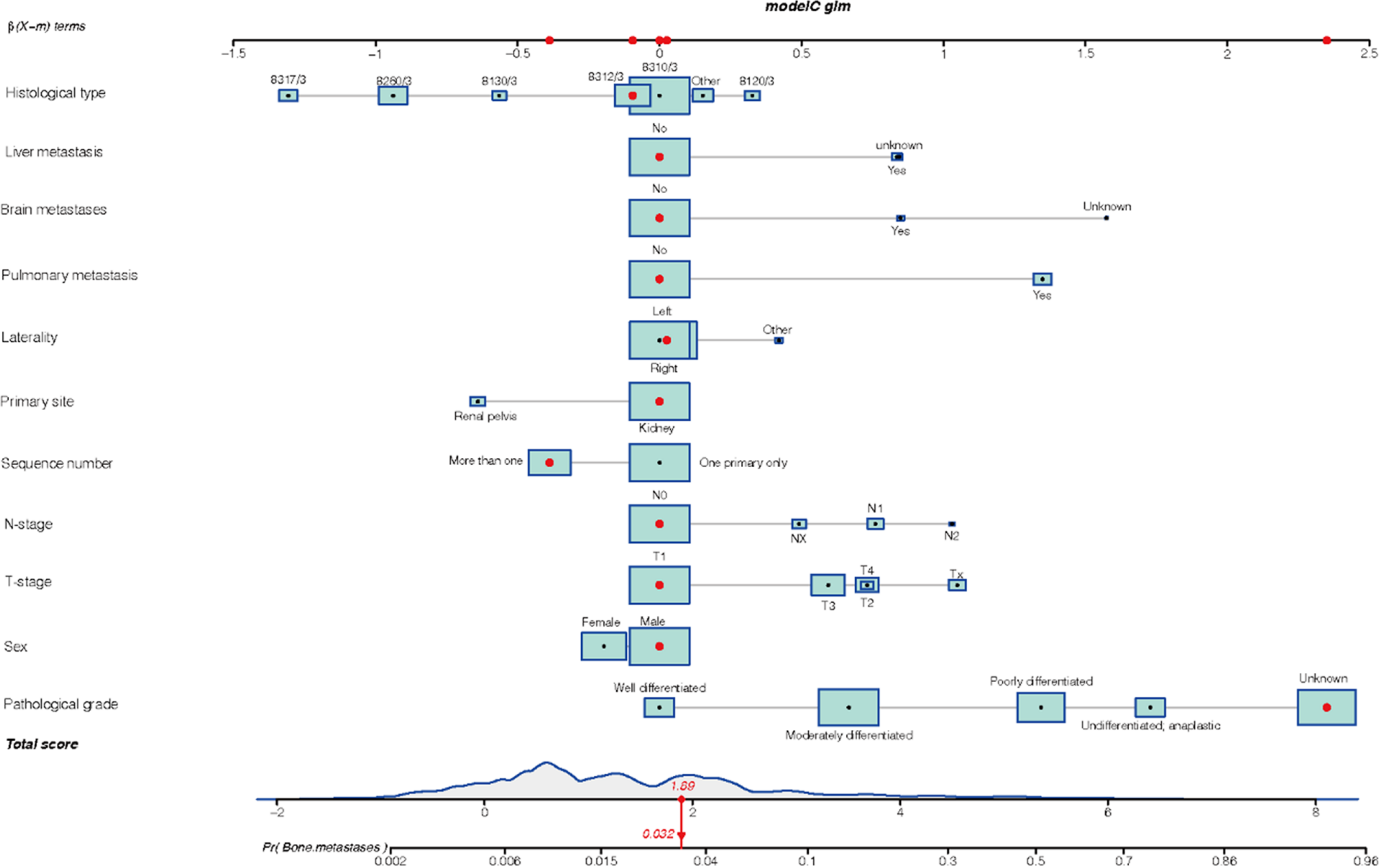
The nomogram model of bone metastasis in patients with kidney cancer: 8317/3 represents chromophobe renal cell carcinoma, 8260/3 represents papillary adenocarcinoma, 8130/3 represents papillary transitional cell carcinoma, 8312/3 represents renal cell carcinoma, 8310/3 represents clear cell adenocarcinoma, 8120/3 represents transitional cell carcinoma, and other represents the number of patients is less than 1,000.

Statistically significant variables demonstrated by regression analysis were used to develop the nomogram. **Figure 1** illustrated the nomogram for the risk of BMs in mRCC incorporating multiple clinical factors. In our nomogram, the effect of the variables on the endpoint events was reflected in the respective line lengths and corresponding scores. Different patients had individualized scores. The total score associated with each variable constituted the probability that the patient will develop BMs.

**Figure 2:**
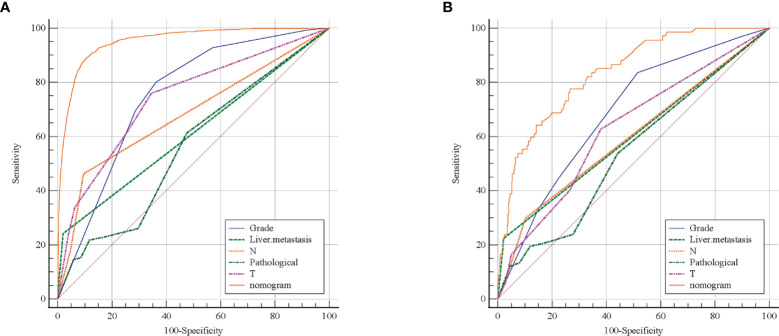
Receiver operating characteristic (ROC) curve evaluated the performance of predictive model. The training group **(A)** and external validation group **(B)** showed the nomogram has better predictive performance than any single variable.

**Figure 3:**
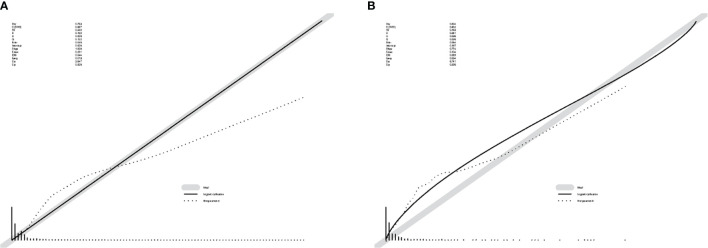
Calibration plots of the nomogram. **(A)** The calibration plots for the training group. **(B)** The calibration plots for the validation group. These curves show the correlation between the predicted probability (*x*-axis) and the actual incidence of the event (*y*-axis).

**Figure 4:**
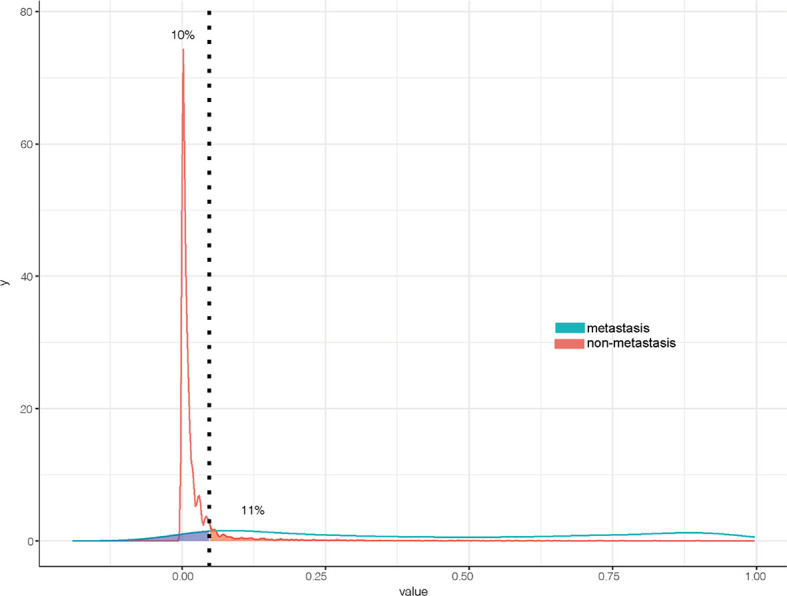
Probability density functions of the predictive models.

**Figure 5:**
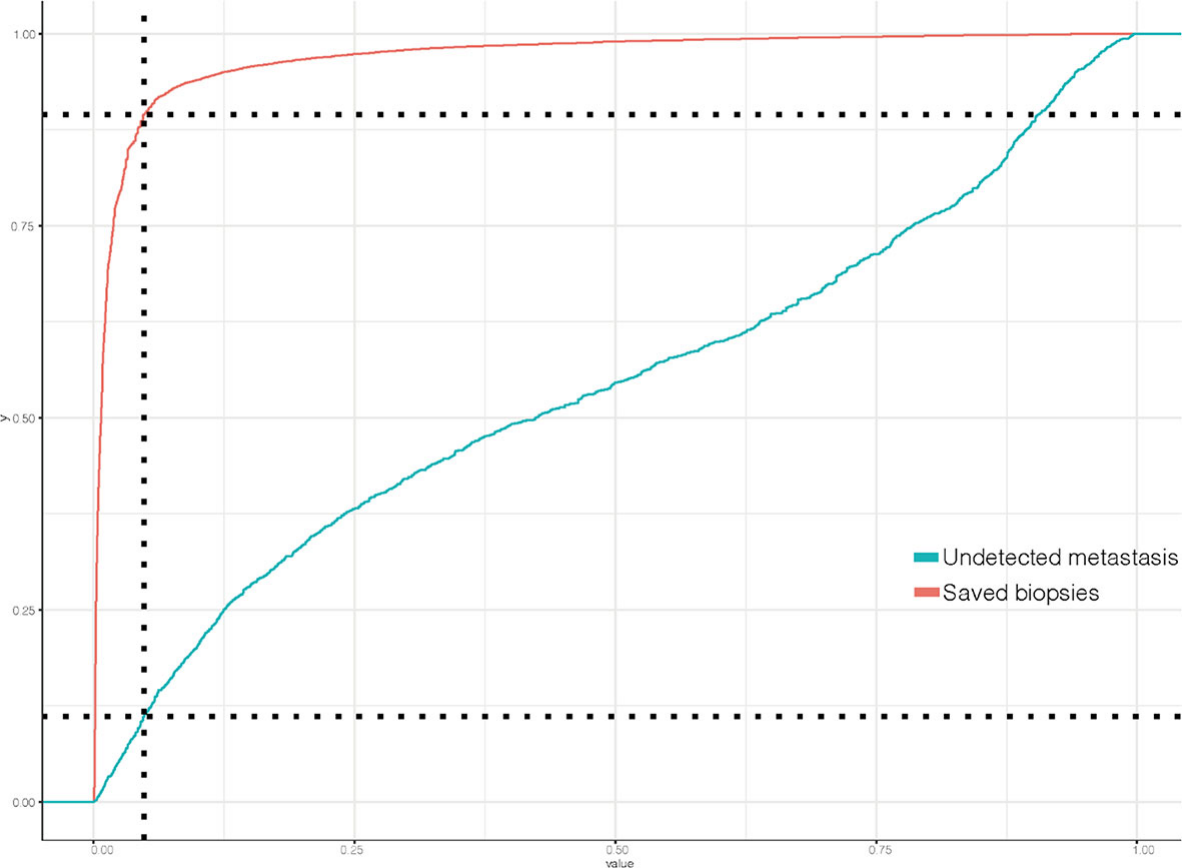
Clinical utility curves of the predictive model.

**Figure 6:**
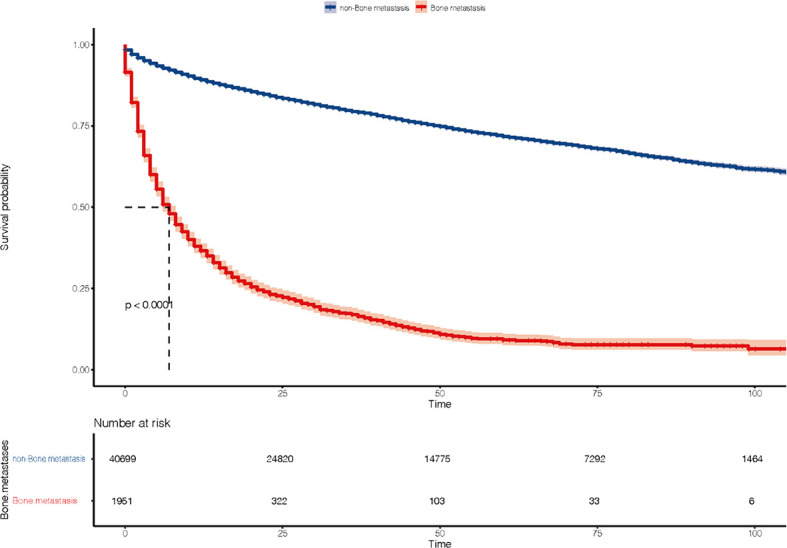
Kaplan-Meier survival curve of bone metastases in patients with kidney cancer.

The ROC curve (**Figure 2**) was used to assess the predictive performance of the nomogram, and the AUC of the training group (AUC = 0.952) and the validation group (AUC = 0.836) showed that the model was useful for superior predictive ability (**Table 4**). Meanwhile, the calibration plots of the training and validation groups were also used to evaluate the accuracy of the nomogram prediction results with respect to the actual occurrence. Ideally, the calibration curve is a diagonal line; at this time, the predicted probability is equal to the true probability. The calibration curves of our nomogram confirm the good agreement between the actual and predicted values in **Figure 3**. As shown in **Figure 4**, the PDF for nonmetastatic patients is concentrated in the portion representing 0%–10% risk of metastasis, while the distribution of the curve for metastatic patients is relatively flat. **Figure 5** shows the percentage of patients with undetected metastases and preserved biopsies detected at any probability threshold and suggests 5% as the threshold probability for making a clinical decision. In addition, the Kaplan-Meier curve for cases that underwent risk stratification confirmed a significant survival advantage for patients without BMs (**Figure 6**). This discovery is one solid evidence to confirm the significance of our study. When using the proposed web calculator, the corresponding BM risk scores could be obtained by selecting several risk factors confirmed in this study (https://liwenle0910.shinyapps.io/RCCapp/).

**Table 4:** The area under curve (AUC) for predicting BMs.

Variables	The training group			The validation group		
	AUC	SE	95% CI	AUC	SE	95% CI
Grade	0.752	0.00404	0.748–0.756	0.686	0.0305	0.654–0.717
Histology	0.558	0.00568	0.553–0.563	0.541	0.0341	0.507–0.575
T stage	0.741	0.00528	0.737–0.745	0.628	0.0334	0.595–0.660
N stage	0.681	0.00519	0.677–0.686	0.598	0.0286	0.564–0.631
Liver metastasis	0.611	0.00446	0.607–0.616	0.601	0.0258	0.567–0.634
Nomogram	0.952	0.00210	0.950–0.954	0.836	0.0239	0.809–0.860

SE, standard error; 95% CI, 95%, confidence interval.

## Discussion

Similar to other solid tumors, bone metastasis is often associated with the progression of metastatic kidney cancer. Interestingly, Becerra and colleagues (18) found that the mutated genes were not consistent in the primary tumor and metastatic samples, enlightening us that metastasis may possess genetic characteristics that are distinct from the primary one. Meanwhile, previous reports confirmed that BMs was an independent risk factor for mRCC prognosis (5, 8, 14, 15). Thus, identifying patients at high risk for BMs provides a sturdy basis for guiding treatment (timing and procedures of surgery, chemotherapy, and radiation therapy).

In this study, a nomogram of risk factors for BMs was developed. Eleven risk factors were identified, including sex, pathological grade, T-stage, N-stage, sequence number, brain metastases, liver metastasis, pulmonary metastasis, histological type, primary site, and laterality. In addition, the ROC curves and calibration curves were used to demonstrate favorable discrimination and calibration plots. The use of combinatorial lines simplified the patient status and provided a visual assessment on the nomogram as a total score (15, 19).

Gender-associated genetic specification to mRCC has also been reported previously, involving multiple risk genes including 14q24.2 (DPF3) and 2p21 (EPAS1) (20). Meanwhile, the impact of sex on RCC-specific mortality is inconsistence, as lower RCC-specific mortality was detected in premenopausal women than in men of the same age, but the difference diminished after menopause (21). Chen et al. (22) reported a distinct gender bias for RCC, with significantly higher prevalence (62.6%) and BMs (76.3%) in male, but they were unable to confirm the correlation between gender and BMs. Interestingly, another paper based on the SEER database concluded that male patients were more likely to develop BMs (23). Reasons for the disagreement may include the fact that the former is a single-center retrospective study. In Chen’s study, the study time spanned at 16 years in order to recruit a sufficient number of subjects, resulting in the variant diagnostic criteria, given the development in early and precise diagnosis of RCC. Our study confirmed a strong correlation between BMs and male RCC patients due to the high risk of RCC invasion in men. Women more frequently use healthcare system, including the scheduled abdominal radiological examination, resulting in early detection, which may further contribute to the difference of BMs in RCC (24).

Variables including pathological grade, T-stage, and N-stage associated with patient overall survival were also shown to be related to BMs (19, 24–26). A possible explication for the higher risk of BMs in mRCC patients with high-level pathological grade and advanced T-stage and N-stage could be the possibility to possess drastic aggressiveness. The skeleton-specific microenvironment has been found to be a suitable “soil” for the growth of mRCC. The invasion of cancer cells requires roughly three processes: “escape” (malignant cells leave the kidney), “metastasis” (reaching the skeletal microenvironment suitable for mRCC growth *via* blood vessels), and “colonization” (formation of new lesions in the involved sites) (6). The metastasis tumor cells will invade the blood vessels and colonize in the target bone through a characteristic preference (27, 28). Thus, predictors suggesting high malignancy of RCC still had efficacy in assessing BMs. Pulmonary/brain/liver metastasis are also predictors for the evaluation of BMs compared with patients without multiple metastasis. As mentioned above for the metastatic mechanism, the renal vein and inferior vena cava are a critical part of distant metastasis. In patients developing pulmonary/cerebral/liver metastasis, tumor cells have escaped and the risk of BMs is inevitably significantly elevated (29–32). The KM curves presented in this study also confirmed that patients with BMs have a worse prognosis than patients without.

As demonstrated in our nomogram, of all the histologic types that are available from the SEER database, the most common and rarest subtypes of BMs are transitional cell carcinoma (TCC) and chromophobe RCC, respectively. TCC is a relatively rare renal malignancy that accounts for approximately 10% of all genitourinary cancers (33, 34). Its histologic feature has been shown to resemble bladder cancer, with a 5-year survival rate of 77%–80% in T1 patients and a highest risk of BMs (33, 35). For comparison, the metastatic inertia of chromophobe RCC is consistent with previous studies. Since Thoenes separated chromophobe RCC from RCC three decades ago, substantial evidence demonstrated its 10-year OS is 80%–90% and metastasis rate is only 5% which supports the definition of chromophobe RCC as a low malignant tumor (36–38).

The debate about the laterality has not reached a consensus until now. As we have known, the left renal vein has more vascular collateral circulation, which brings together multiple veins of the lumbar region and may induce more metastasis. Therefore, the left tumors may develop more metastasis than those on the right side (39). This idea is also reflected in the report of Morri et al. (40) Owing to the concern for laterality, surgeons usually remove left lesions as possible, which leads to more negative margins being observed in left-sided cases. However, we did not find that the difference between the left and right sides significantly affected the BMs. Conversely, patients with bilateral/other types were more likely to develop BMs. This finding may be associated with hereditary RCC, which is primarily characterized by bilateral or multifocal masses. Hereditary RCC frequently presents with perirenal fatty infiltration and renal vein infiltration; retroperitoneal and mediastinal lymph nodes, liver, and bone are common targets of metastasis (41, 42). Another risk factor indicating the relationship between tumor location and BMs is the primary site. Renal pelvic RCC has a lower risk of BMs. In this regard, we believe the following explanation is acceptable. On the one hand, the clinical symptoms of renal pelvis tumors are obvious, 80% of patients will develop hematuria, and early medical consultation can effectively control the carcinoma progression. On the other hand, the surgical criteria for renal pelvic RCC require a greater extent of resection compared with kidney cancer, which is one of the explanations for effective patient protection (43). However, previous studies have reported the renal vein or inferior vena cava (IVC) involvement is associated with early onset of metastasis in renal pelvis RCC (29). There is a long way to go for exploring the mechanisms between the site of carcinoma origin and BMs.

Furthermore, the extensive overlap of risk factors for prognosis and BMs often leads to confusion between urologists and orthopedic surgeons about this concept. Prognostic factors typically indicate that the association between patient status and survival for patients eligible for this variable does not depend on the treatment regimen received (44). The findings of this study could not simply equate severe patients with patients with metastasis unless BMs were the direct cause of the patient’s death. “Severe” is often used to define a patient’s overall health status rather than simply describing the tumor, especially for patients with BMs who were vulnerable to the threat of adverse skeletal events and an increasing financial burden (45, 46).

Our study demonstrated for the first time that sequence number was associated with BMs in patients with RCC. As the criteria presented previously, we found that patients with >1 primary tumor were less likely to develop BMs, possibly due to the poorer prognosis and shorter survival of patients with multiple tumors, resulting in the lack of necessary time for BMs to form (45).

Compared with other tumors, RCC is characterized by high vascularity, which poses a serious challenge for treatment. Vascular endothelial growth factor (VEGF) plays an important role in promoting angiogenesis in RCC. As a result, targeted therapies represented by VEGF inhibitors (bevacizumab, sunitinib, axitinib), as first-line treatment for advance patients, have been developed, showing impressive clinical outcome (4, 47, 48). Bone-targeted treatment (bisphosphonate) and surgical intervention are also effective to treat mRCC with BMs. Surgical recommendations for early radical resection of the primary tumor and/or skeletal lesions have been shown to help prolong patient survival. Benefiting from this, earlier detection and higher surgical rates led to a better prognosis for patients with mRCC metastasizing to the long bones (8). Notably, the high vascularity can result in the devastating bleeding during procedure without proper pretreatment. Several studies have reported that preoperative embolization has shown significant benefits in reducing perioperative blood loss in mRCC patients (49–51). Thus, the developed predictive model will be useful in risk stratification, surveillance of cases, decision treatment, prolongation of survival, and control of spending.

Unlike conventional logistic regression analysis that simply suggests parameters affecting BMs in mRCC (23), our proposed prediction model presented as a nomogram was able to quantify these predictors by scoring each risk factor. Higher scores indicated increased risk of developing BMs. In addition, we offered clinicians an online web calculator that fits the digital definition. By clicking on the link below and typing in the patient’s personalized information, users can quickly obtain the target BMs risk (https://liwenle0910.shinyapps.io/RCCapp/).

Nevertheless, there were several limitations in our study. First, this article is a retrospective cohort study, and artificial selection bias may have an adverse effect on the conclusion. Secondly, given that the variables recorded in the SEER database are stereotyped, some valuable clinical predictors were not involved in this study, including common tumor markers such as AFP, CE-199, molecular susceptibility, and the Fürhman classification (22, 52). Meanwhile, we extracted the cases according to ICD-O codes, not the latest published WHO histological types. Notably, this population-based study included an adequate number of patients, which ensures the credibility of the conclusions. Future studies need to go further to incorporate tumor characteristics, laboratory results, and treatment regimens to establish a higher dimensional predictive model.

## Conclusion

We retrospectively investigated the risk factors impacting the appearance of BMs in kidney cancer. Combining the SEER database and an independent external validation dataset, this study proposed and validated a prediction model that incorporated sex, pathological grade, T-stage, N-stage, sequence number, brain metastases, liver metastasis, pulmonary metastasis, histological type, primary site, and laterality. The adverse prognosis of BM patients was confirmed *via* KM curve. The composite predictive tool consisting of nomogram and web calculator provides important consideration for the multidisciplinary management.

## Data Availability Statement

The original contributions presented in the study are included in the article/supplementary material. Further inquiries can be directed to the corresponding authors.

## Ethics Statement

This study is based on the SEER database and does not require ethical approval.

## Author Contributions

SD and HY completed the study design. SD, HY, Z-RT, and YK performed the study and collected and analyzed the data. SD and HY drafted the manuscript. HW, KT, and WL provided the expert consultations and suggestions. SD, KT, and WL conceived of the study, participated in its design and coordination, and helped to embellish language. All authors contributed to the article and approved the submitted version.

## Funding

This work is supported by the National Natural Science Foundation of China (No. 81601901) and Natural Science Foundation of Liaoning, China (No. 2019-MS-079).

## Conflict of Interest

The authors declare that the research was conducted in the absence of any commercial or financial relationships that could be construed as potential conflict of interest.

## Publisher’s Note

All claims expressed in this article are solely those of the authors and do not necessarily represent those of their affiliated organizations, or those of the publisher, the editors and the reviewers. Any product that may be evaluated in this article, or claim that may be made by its manufacturer, is not guaranteed or endorsed by the publisher.
